# Subepithelial Lesions of the Ocular Surface: A Review

**DOI:** 10.1007/s40135-025-00335-8

**Published:** 2025-06-21

**Authors:** Wisam Najdawi, Wendy J. Li, Sofia De Arrigunaga, Mona M. Amer, Anat Galor, Carol L. Karp

**Affiliations:** 1 Department of Ophthalmology, Bascom Palmer Eye Institute, University of Miami, 900 NW 17th St, Miami, FL 33136 USA; 2 Department of Ophthalmology, Miami Veterans Administration Medical Center, Miami, FL USA

**Keywords:** High-resolution optical coherence tomography, Conjunctival melanoma, Conjunctival lymphoma, Conjunctival myxoma, Conjunctival neuroma, Benign reactive lymphoid hyperplasia, Conjunctival amyloidosis

## Abstract

**Purpose of Review:**

Subepithelial lesions of the ocular surface represent a diverse group of pathologies which may be difficult to diagnose clinically. Some of these lesions are relatively uncommon, may result in systemic manifestations, or occur secondary to systemic disease. The purpose of this review is to summarize current approaches to the diagnosis and management of six subepithelial conjunctival lesions.

**Recent Findings:**

The standard for the diagnosis of subconjunctival lesions remains histopathologic evaluation; however, high-resolution anterior segment optical coherence tomography (HR-OCT) is a useful supplemental diagnostic tool that may facilitate diagnosis. Recent advancements in the management of subconjunctival lesions include targeted systemic therapies in conjunctival melanoma and ultra-low dose radiation radiotherapy in conjunctival lymphoma.

**Summary:**

The development of HR-OCT has provided clinicians with valuable supplemental diagnostic information to guide the diagnosis of subepithelial lesions. Additionally, novel treatment modalities may provide an alternative to traditional surgical interventions in some pathologies.

## Introduction

Lesions of the conjunctiva encompass a diverse range of pathologies and may be classified as epithelial, subepithelial, or both based on their location. Clinically, the etiologies of subconjunctival lesions are diverse, and they can be challenging to diagnose. It is important to differentiate these lesions, as some occur as primary tumors with resulting systemic ramifications and others occur as manifestations of systemic disease. Malignant subepithelial lesions include entities such as melanoma and lymphoma, and benign lesions include reactive lymphoid hyperplasia, myxomas, neuromas, and amyloidosis, among others. The severity of these pathologies can range from benign to potentially sight- or life-threatening, and differentiating these subepithelial lesions can present a challenge even among experienced clinicians, as some lesions are relatively uncommon and some present similarly. As such, prompt and accurate diagnosis and management are critical for ensuring favorable patient outcomes.

In addition to clinical examination, histopathologic evaluation remains the clinical standard for the diagnosis and staging of subepithelial conjunctival lesions and is ultimately required for definitive diagnosis in many cases. High-resolution anterior segment optical coherence tomography (HR-OCT) has proven to be an invaluable imaging modality for the evaluation and management of ocular surface lesions such as ocular surface squamous neoplasia. Similarly, it can complement the clinic examination and histopathologic evaluation to guide clinicians in diagnosing subepithelial conjunctival lesions [1]. HR-OCT utilizes spectral-domain technology with short wavelength light to rapidly generate in vivo cross-sectional images of the ocular surface and anterior segment at resolutions as low as 5 microns [2]. This imaging modality is non-invasive and provides useful adjunct information that may aid clinicians in differentiating subepithelial lesions.

In this review, we present the clinical and histopathologic findings of six subepithelial lesions, including malignant tumors (conjunctival melanoma [CM] and conjunctival lymphoma [CL]) and benign tumors (benign reactive lymphoid hyperplasia [BRLH], conjunctival myxoma [CMx], conjunctival neuroma [CN], and conjunctival amyloidosis [CA]). Additionally, we highlight how HR-OCT can inform clinical decision making and briefly review current management practices for each pathology.

## Conjunctival Melanoma

### Disease Entity

CM is a potentially life-threatening malignancy originating from atypical melanocytes in the basal layer of the conjunctival epithelium and ultimately extending into the underlying substantia propria. CM arises from primary acquired melanosis (PAM) in 53–80% of cases, de novo in 15–37% of cases, and from conjunctival nevi in 4–9% of cases [3–6]. The risk of progression from PAM to CM is 0% with no or mild atypia and 13–48% in the presence of severe atypia [7, 8]. The risk of progression from a conjunctival nevus to CM is 0.7% [9].

### Epidemiology

CM is the second most prevalent malignancy of the conjunctiva [10]. It accounts for 0.25% of all melanomas and 4.8% of ocular melanomas [11]. In general, the incidence of CM has increased over time, and reported incidence rates range from 0.3 to 0.8 per million individuals per year with regional variation [12]. Since 2000, the crude incidence of CM in the United States has increased by approximately 18% from 0.33 per million individuals to 0.39 per million individuals in 2022, impacting an estimated 2390 people nationwide [13]. A 2020 study inclusive of 41 European cancer registries reported an incidence of CM of 0.46 per million individuals [14]. CM occurs equally among men and women [13, 14]. The mean age of diagnosis is 62 years, and incidence increases with age [6, 13–15]. CM incidence varies with ethnicity, occurring most frequently in Whites and least frequently in Blacks (RR = 2.72) [13, 16].

### Risk Factors

Unlike with cutaneous melanoma, the role of ultraviolet (UV) radiation in the development of CM is unclear [17, 18]. While the incidence of CM has been shown to increase with decreasing latitudes, additional factors such as skin type and genetics may play a significant role [19]. Individuals with fair skin are at greater risk for the development of CM, with up to 85% of CM patients demonstrating Fitzpatrick skin types I or II [4]. Genetic abnormalities also play a role in the development of CM. *BRAF* mutations are observed in 35% of patients with CM, and the most common mutation occurs in V600E accounting for approximately 80% of all *BRAF* positive mutations [20]. *BRAF* mutations are an early event in the tumor growth process and occur more frequently in younger male patients who have lesions with a sun-exposed location, mixed or absent pigmentation, or a nevus origin [20]. In addition, mutations in the *NF1* gene are observed in 33% of patients and result in MAPK pathway activation [21]. In a cohort of 101 patients, 26% exhibited mutations in the *NRAS* gene and 25% had mutations in the *ATRX* gene [22]. Less common mutations that may be associated with CM include mutations in *KIT* and *TERT* [22, 23]. Though *ATRX* and *KIT* mutations may also occur in mucosal melanomas, CM is generally thought to exhibit a different genetic profile that more closely resembles cutaneous melanoma than mucosal melanoma [24].

### Clinical Presentation

**Fig. 1:**
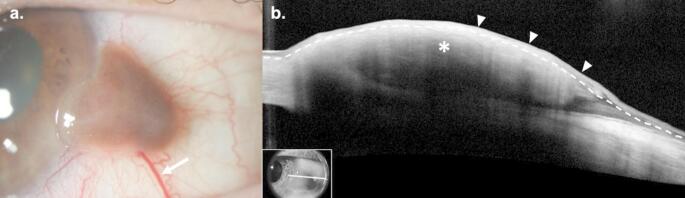
Conjunctival Melanoma. (**a**) Slit lamp examination of the left eye of a 68-year-old White female revealed an elevated, gelatinous, brown conjunctival lesion at 3:00 with an associated feeder vessel (arrow). (**b**) High resolution optical coherence tomography (HR-OCT) revealed a hyperreflective subepithelial lesion (asterisk) with overlying hyperreflective epithelium (above dashed line) of normal thickness (arrowheads). Findings were consistent with, and biopsy confirmed the diagnosis of malignant melanoma. Inset shows scan location

CMs typically present as a thickened, elevated, pigmented lesion with surrounding melanosis (Fig. 1a) [12, 25]. CM almost always occurs unilaterally and involves the bulbar conjunctiva in 92% of cases [26]. The corneoscleral limbus is involved in 48–61% of cases [25]. CM occurs most frequently in the interpalpebral conjunctiva; however, lesions can uncommonly occur in the palpebral, tarsal, and forniceal conjunctiva, plica, or caruncle [6, 25]. In pigmented lesions, coloration ranges from light brown to dark brown to, rarely, black [12, 27]. Lesions may be amelanotic in 11–19% of cases or demonstrate partial/mixed pigmentation in up to 36% of cases [5, 6, 28]. As such, it is critical to maintain a high index of suspicion for CM for all subconjunctival lesions regardless of pigmentation until the diagnosis can be confirmed or ruled out. Feeder vessels are present in 39% of lesions [5]. The most common presenting symptom is notice of a pigmented or fleshy spot; however, less frequently, lesions may be associated with foreign body sensation, ocular pain, and irritation [25].

### HR-OCT

HR-OCT of CM lesions demonstrate an elevated, hyperreflective subepithelial mass (Fig. 1b). The overlying epithelium is normal to slightly thickened with variable hyperreflectivity of the basal layer, suggesting the involvement of atypical melanocytes. In the case of larger lesions, visualization of the posterior border and internal structural detail may be limited due to posterior shadowing [29–31]. CM can be distinguished from precursor lesions (i.e. PAM and conjunctival nevi) on HR-OCT as PAM demonstrates no subepithelial component and conjunctival nevi often demonstrate internal cystic structures [32, 33]. The findings on HR-OCT, namely of subepithelial hyperreflectivity, are especially helpful in cases of amelanotic CM where the diagnosis is clinically unclear.

### Diagnosis

Features such as pigmentation, nodularity, changes in size, prominent feeder vessels, or involvement of unusual locations (i.e., caruncle, tarsal, or forniceal conjunctiva), particularly in preexisting areas of PAM, may indicate a conjunctival malignant process. HR-OCT can be a helpful tool to guide the physician, especially in clinically challenging cases. Once CM is suspected, the diagnosis must be confirmed via histopathologic analysis. An excisional biopsy is preferred to minimize the risk of tumor seeding. Histopathologic evaluation of CM can be difficult even among experienced pathologists as evaluation requires greater reliance on cytologic features than standard architectural patterns [27]. Grossly, tumor size ≥ 4 mm and the presence of ulceration impart greater risk of local metastases, distant metastases, and disease-specific death [34, 35]. Four types of atypical melanocytes, including spindle cells, balloon cells, epithelioid cells, and small polyhedral cells, may be present [25–27]. In each cell type, the presence of atypia (e.g. large nuclei, prominent nucleoli, and mitotic activity) is concerning for malignancy [27]. Tumors composed primarily of small polyhedral cells can be more difficult to assess and may require recognition of unique architectural patterns, including intraepithelial pagetoid growth, radial intraepithelial extension beyond the edge of the subepithelial lesion, patchy or bandlike inflammation at the basal layer of the lesion, immature cells at the base of the lesion, and invasion of the cornea or sclera [25–27, 36]. Patients with positive sentinel lymph node biopsies are at greater risk for distant metastases and disease-specific death [34]. Immunohistochemistry plays a limited role in the diagnosis of CM, though melanocytic markers such as HMB-45, S100, SOX-10, MART-1, and melan-A may provide useful information regarding architectural features and melanocytic density [26, 27, 37, 38]. As described below, markers for immune signaling molecules such as PD-1, PD-L1, CTLA-4, and LAG-3 may be useful in characterizing CM.

### Management

The primary treatment for CM is surgical excision with wide 3–4 mm margins utilizing a “no-touch” technique and cryotherapy applied at the margins in a double freeze, slow thaw manner [39]. Partial sclerectomy and alcohol epitheliectomy can be performed to ensure tumor-free margins in lesions that invade the sclera and encroach onto the cornea, respectively [25, 26]. A dry surgical field should be maintained during tumor excision and fresh instruments should be utilized for subsequent steps of the procedure to prevent tumor seeding [25, 40]. The size of the lesion determines the method of closure. Primary closure of Tenon’s fascia and the conjunctiva can be used in small lesions [26]. Amniotic membrane can be used to aid with closure in larger lesions [41]. Local adjuvant therapies, including topical chemotherapy with mitomycin C (MMC), topical immunotherapy with interferon alpha-2b, brachytherapy, external beam radiotherapy (EBRT), and proton-beam radiotherapy, may be employed at variable time intervals to reduce recurrence risk [25, 42]. For example, one or two cycles of adjuvant MMC can be considered once epithelialization is complete post-surgery. Topical chemotherapy and radiotherapy, while useful adjuvants, are generally not used as the primary treatment of CM [25, 26]. Widespread CM necessitates a more extensive approach that combines surgery, brachytherapy, and potential orbital exenteration. Patients with CM should be referred to a medical oncologist for systemic workup to rule out metastatic disease. Whole-body positron emission tomography/computed tomography (PET/CT), rather than the standard “eye to thigh” scans, may be employed to detect metastases [25].

Recent investigations have identified potential signaling pathways and immune cells that may serve as targets for CM systemic therapies. Checkpoint inhibitors (CPI) are medications that target key immune checkpoint molecules such as PD-1, PD-L1, CTLA-4, and LAG-3. PD-1, expressed on T cells, and its ligand PD-L1, often expressed on cancer cells, interact to suppress T cell activity, while CTLA-4 and LAG-3 inhibit T cell activation in the early stages of immune responses. At the time of writing, five CPIs have been approved for use in locally advanced and metastatic melanoma by the Federal Drug Administration, including ipilimumab (2011), nivolumab (2014), pembrolizumab (2014), relatlimab/nivolumab (2022), and atezolizumab (2024). Nivolumab and pembrolizumab target PD-1, atezolizumab targets PD-L1, ipilimumab targets CTLA-4, and relatlimab targets LAG-3. These medications have increasingly been utilized in the treatment of cutaneous melanoma. Given the similar biological and molecular features shared by cutaneous and conjunctival melanoma, it is hypothesized that CPI could be an effective treatment option for CM [43, 44]. The literature is limited to few case reports documenting unresectable or metastatic CMs that have demonstrated partial to complete regression with CPI [45–51]. No prospective studies investigating CPI in the management of CM exist to date. These targeted therapies are promising, and large-scale studies are needed to assess safety and efficacy.

### Prognosis

The reported 6-year recurrence rate of CM is 33% with factors predictive of recurrence including older age and more advanced T category [52]. The reported 5-year and 10-year metastasis rates of CM are between 9 and 16% and 22%, respectively, with the most common site of metastasis being the regional lymph nodes (cervical, preauricular, postauricular, and submandibular) [5, 15, 53, 54]. Systemic metastases are less common; however, the most common sites of systemic involvement include the liver (45–46%), lungs (38–45%), brain (10–13%), and bone (8–10%) [15, 53, 54]. Each incremental rise in T category carries an 89% increase in risk for the development of metastases [53]. Tumors arising de novo are thought to carry greater risk of metastasis and disease-specific mortality; however, the literature is mixed [3, 6]. The reported 5-year survival rate of CM ranges from 74 to 93%, though cumulative mortality rates vary with tumor stage [5, 11, 55, 56]. The cumulative survival rate decreases to 58% in patients who have developed systemic metastases [53].

## Conjunctival Lymphoma

### Disease Entity

CL is a malignant tumor arising from the clonal proliferation of lymphocytes in the subepithelial space. CLs are predominantly extranodal marginal zone B-cell lymphoma (EMZL) of mucosa-associated lymphoid tissue (MALT), which accounts for 81% of cases. Additional subtypes of CL include follicular (8%), diffuse large B-cell (3%), mantle cell (3%), and, rarely, T-cell lymphoma (2%). CL accounts for only 5–10% of all extranodal lymphomas and can be a primary, well-defined tumor or a secondary manifestation of systemic lymphoma [57].

### Epidemiology

CL is the third most common malignancy of the ocular surface, following squamous cell carcinoma and CM [58]. In a review of 1014 conjunctival lymphoid neoplasms, 98% were found to be of B-cell lineage [57]. In 1168 patients with ocular adnexal lymphoma, 97% were of B-cell origin [59]. Another large-scale study of 17,878 patients with ocular and orbital lymphomas revealed that non-Hodgkin B-cell lymphoma was the most prevalent subtype, accounting for 85.4% of cases [60]. EMZL occurs more often in women and, like follicular lymphoma (FL), usually presents in the seventh decade of life. The rarer diffuse large B-cell lymphoma (DLBCL) and mantle cell lymphoma (MCL) have a predilection for the male sex and typically present in the eighth decade of life [57]. Unlike EMZL and FL, DLBCL and MCL are frequently secondary diseases [61]. MCL is characterized by bilateral presentation and a high frequency of stage IVE secondary disease according to the Ann Arbor staging classification [61].

Although CL typically occurs in middle-aged adults, it can also affect children, though this is extremely rare, and its incidence is unknown. Current understanding of CL in pediatric populations remains limited to case reports and small case series. In a retrospective series of 55 pediatric patients with ocular adnexal lymphoma, EMZL was found to be the most frequent subtype of CL. Burkitt lymphoma and T-cell lymphoma were found in higher proportions compared to the adult population, while FL was less common [62].

### Risk Factors

Predisposing factors linked to the development of CL include immune dysfunction, autoimmune conditions (Sjogren’s syndrome, IgG4 disease, systemic lupus erythematosus, Hashimoto’s thyroiditis), infectious etiologies, and genetic mutations [63–66]. CL has been correlated to chronic inflammation of the conjunctiva triggered by antigens, resulting in the sustained accumulation, proliferation, and genetic alterations of monoclonal B and T lymphocyte populations. Inflammatory mediators may further stimulate the growth and survival of abnormal lymphoid cells. In terms of infectious etiologies, *Helicobacter pylori* infection is widely acknowledged as a key cause of gastric MALT lymphoma. *H. pylori* and *Chlamydia psittaci* are potential co-factors in CL, but their role remains controversial [67–71]. The conjunctival microbiota of lymphoma patients has been shown to be compositionally different from that of healthy controls, and it has been postulated that this dysbiosis in patients with EMZL may contribute to its pathogenesis [72, 73]. BRLH is believed to be a potential precursor to CL in younger patients, although the data supporting this hypothesis is limited [74, 75]. Mutations in tumor suppressors, transcription regulators, and chromatin remodeling genes have been associated with various subtypes of CL [63].

### Clinical Presentation

**Fig. 2:**
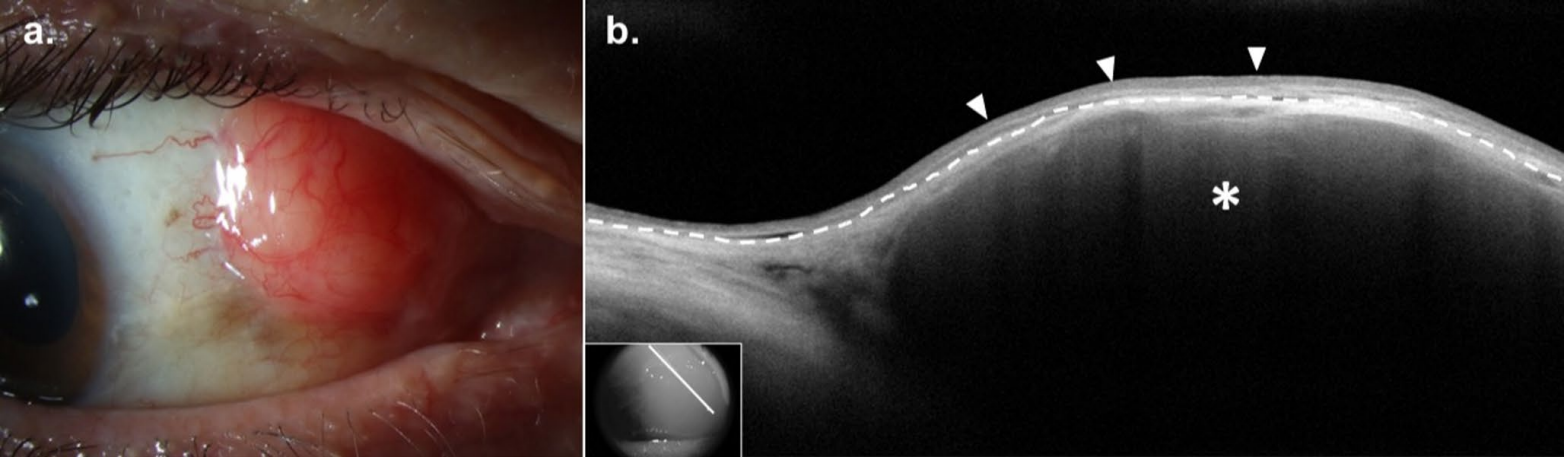
Conjunctival Lymphoma. (**a**) Slit lamp examination of the right eye of a 77-year-old White male revealed a superonasal, highly elevated “salmon patch” colored subepithelial conjunctival lesion. (**b**) High resolution optical coherence tomography (HR-OCT) revealed a subepithelial, homogeneous, hyporeflective infiltrate (asterisk) and a normal overlying epithelium (arrowheads). The border between the epithelium and subepithelial space is labeled (dashed line). Findings were consistent with, and biopsy confirmed the diagnosis of follicular lymphoma. Inset shows scan location

CL typically presents as a unilateral or bilateral, painless, “salmon patch” subepithelial lesion on the conjunctiva, sometimes with feeder vessels (Fig. 2a). Alternatively, it may resemble follicular conjunctivitis. Locations of CL include the conjunctival fornix (44%), midbulbar region (42%), caruncle (7%) or limbus (7%) [76]. It is typically oval-shaped when occurring on the bulbar conjunctiva and oblong in the fornices, conforming to the contour of the fornix. Symptoms include a mass, irritation, ptosis, epiphora, blurry vision, proptosis, diplopia, or, occasionally, no symptoms. CL can extend to the orbit, eyelid, or uvea. It can manifest as a primary lymphoma that is confined to the periocular region (EMZL and FL) or as a secondary lymphoma with systemic lymphoid infiltrates (DLBCL and MCL). On slit lamp examination, CL and BRLH often appear similarly, and a biopsy is needed to confirm the diagnosis. Thus, making the diagnosis of CL may be challenging.

### HR-OCT

HR-OCT can be a helpful adjunct tool in the diagnosis of CL. On HR-OCT, CL presents as a hyporeflective, homogenous subepithelial mass with smooth borders composed of monomorphic, stippled, hyporeflective dots (Fig. 2b) [77]. The overlying epithelium is of normal appearance and thickness. Lesions may be bordered superiorly and inferiorly by a hyperreflective band of substantia propria representing conjunctival tissue displaced by the lymphocytic infiltrate. In contrast to CL, BRLH on HR-OCT often reveals a more heterogeneous poorly defined lesion.

### Diagnosis

Clinical examination alone cannot distinguish between benign and malignant lymphoid lesions. Therefore, the diagnosis of CL requires histopathologic evaluation following biopsy of the tumor. Two samples should be taken, one fresh and the other formalin-fixed. The formalin-fixed sample is stained with H&E to evaluate morphology, and the fresh sample is analyzed using flow cytometry for specific immunohistochemical antibodies. CL demonstrates distinct features on histopathological examination. Histologically, it is characterized by a dense lymphocytic infiltrate within the substantia propria. These aggregates often resemble follicular structures, particularly in FL, or diffuse sheets of lymphoid cells. The neoplastic lymphocytes usually exhibit small- to medium-sized cells with round nuclei, scant cytoplasm, and occasional clefting. Immunohistochemical staining commonly reveals a positive reaction for pan-B-cell markers such as CD20 and CD79, alongside variable expression of surface immunoglobulins and the BCL-2 protein. Positive CD3 antibodies are indicative of T-cell lymphomas. The expression of CD5 and CD23 can help differentiate CL from other conjunctival lymphoproliferative disorders, such as BRLH or other non-Hodgkin lymphomas.

### Management

Once CL is diagnosed, a systemic work-up should be completed by a medical oncologist to determine the extent of disease. Work-up incudes a complete blood count, serum chemistries, lactate dehydrogenase, CT or MRI of the orbit, CT scan of other potentially affected areas (i.e. neck, chest, abdomen, pelvis), full body PET scan, and bone marrow aspiration and biopsy [78]. The clinical stage of CL is determined using the Ann Arbor/Lugano staging classification and the American Joint Committee on Cancer Tumor, Node, Metastasis staging system for ocular adnexal lymphoma. The Lugano Classification emphasizes the use of modern imaging, including PET and CT scans, for more accurate staging and monitoring of disease [79]. The gold standard treatment for localized disease is EBRT in doses of 20–30 Gy delivered over 10–20 fractions [80]. Side effects of EBRT at high doses include meibomian gland dysfunction, dry eye, conjunctivitis, early cataract formation, and retinal diseases such as chorioretinopathy, maculopathy, and optic neuropathy [81–83]. Recent studies on ultra-low dose radiotherapy (4 Gy), or “boom-boom” therapy, have demonstrated efficacy and safety in patients with CL [84–87]. In a case series of eight patients, six demonstrated a complete response to boom-boom therapy, two demonstrated a partial response, and no patients demonstrated relapse or required adjunct treatment [88]. In patients with systemic involvement, chemotherapy is utilized. An international cohort study of patients with systemic involvement who were treated with chemotherapy in combination with rituximab showed improved disease-specific survival compared to those treated with chemotherapy alone [85]. This suggests that rituximab-based chemotherapy is the preferred treatment for stage IIIE/IVE EMZL patients. When CL presents bilaterally, systemic treatment may be selected over EBRT, but the ultra-low dose radiotherapy may be selected for these bilateral cases. In addition, antibiotic therapy with doxycycline has emerged as a potential primary treatment for MALT lymphoma, although evidence for efficacy is mixed [89–92]. This approach is based on the possible association between lymphomas and chronic bacterial infections, particularly *C. psittaci*. In a series of 67 patients with CL receiving doxycycline as primary treatment, 37.3% (25/67) of patients achieved a complete response, 11.9% (8/67) a partial response, 44.8% (30/67) had stable disease, and 6.0% (4/67) showed progression. No further progression occurred over 6 years following doxycycline treatment [89]. However, this study is limited by the fact that most patients were not evaluated for *C. psittaci* infection. While a few studies have shown promising results with doxycycline, with partial or complete regression of tumors in some patients, the efficacy of antibiotic treatment varies and remains controversial. Doxycycline may be initiated when CL is suspected due to its low side effect profile and the potential for significant benefit.

### Prognosis

In a study of 263 patients, histological subtype was the main predictor of outcome. EMZL and FL had a good prognosis, with 5-year disease-specific survival rates of 97.0% and 82.0%, respectively. In contrast, MCL and DLBCL had much poorer prognoses, with 5-year disease-specific survival rates of 9.0% and 55.0%, respectively [61]. Age, sex, and Ann Arbor tumor staging are other predictors of survival. Tumor location is also a prognostic factor, with conjunctival EMZL associated with significantly lower all-cause mortality compared to primary eyelid EMZL [59].

## Benign Reactive Lymphoid Hyperplasia

### Disease Entity

BRLH of the conjunctiva is a non-neoplastic condition belonging to the broad spectrum of ocular adnexal lymphoid tumors (OALTs) and is characterized by an abnormal proliferation of lymphoid tissue within the conjunctiva. In a series of 353 patients diagnosed with an OALT, BRLH constituted 23% of cases [93]. BRLH clinically resembles CL, making thorough examination and assessment of suspected lesions essential to rule out malignancy.

### Epidemiology

BRLH typically occurs in older children and young adults, with a slightly higher predilection for the male sex [94]. In a review of 235 cases (54% male and 46% female), the mean age at diagnosis was 35.2 years (range: 5–92 years) [95]. Compared to CL, BRLH occurs in younger patients, usually presenting with an otherwise unremarkable history and without systemic lymphoma.

### Risk Factors

It is postulated that BRLH results from antigenic stimulation of the conjunctival MALT, leading to an immune response that causes the lymphoid tissue to proliferate [96]. BRLH may also be associated with autoimmune disease [97]. The specific risk factors for developing BLRH are unknown.

### Clinical Presentation

**Fig. 3:**
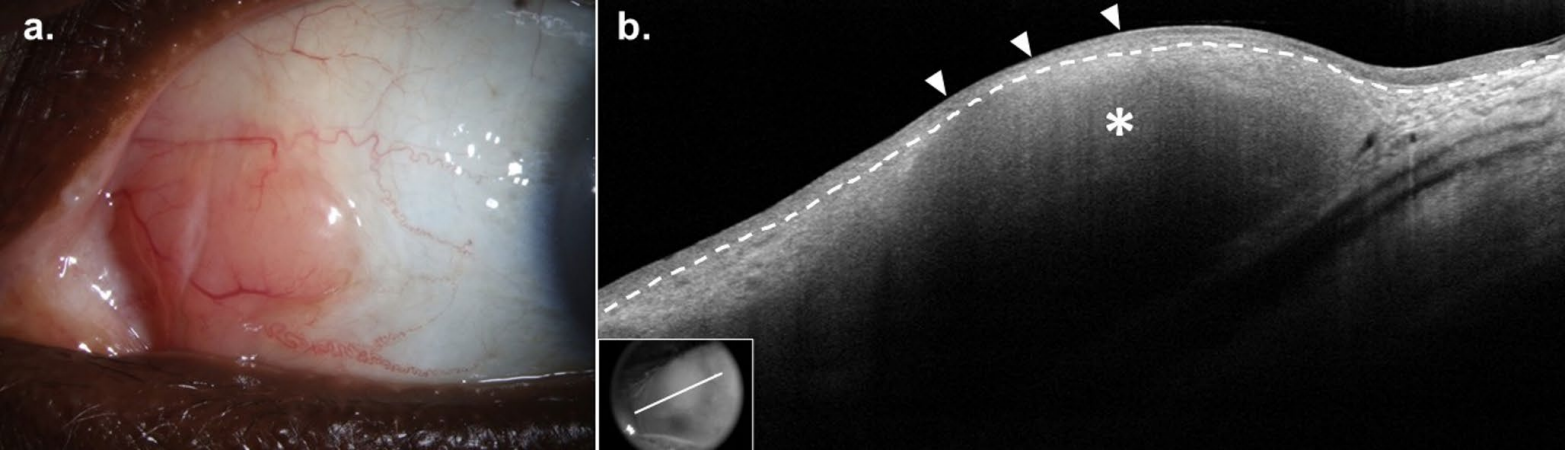
Conjunctival Benign Reactive Lymphoid Hyperplasia. (**a**) Slit lamp examination of the left eye of a 66-year-old Black female revealed a salmon-colored, elevated, nasal subepithelial conjunctival lesion. (**b**) High resolution optical coherence tomography (HR-OCT) revealed a subepithelial lesion (asterisk) with hyporeflective monomorphic infiltrate and a normal overlying epithelium (arrowheads). The border between the epithelium and subepithelial space is labeled (dashed line). Findings were consistent with a benign reactive lymphoid hyperplasia although a biopsy was needed to exclude a lymphoma diagnosis based on HR-OCT features. Biopsy confirmed diagnosis of reactive lymphoid hyperplasia with no morphologic or immunophenotypic evidence of lymphoma. Inset shows scan location

BRLH is indistinguishable from CL clinically. BRLH presents as a slow-growing, salmon-colored lesion on the conjunctiva with no significant neovascularization on slit lamp examination (Fig. 3a). Patients may be asymptomatic or experience enlarged cervical lymph nodes, pain, redness, blurred vision, diplopia, proptosis, or ptosis [95, 98]. Lesions commonly present on the nasal bulbar conjunctiva or fornix [99, 100]. When symptomatic, patients usually complain of a palpable mass on the conjunctiva with associated irritation.

### HR-OCT

Like CL, BRLH presents as a subepithelial lesion on HR-OCT with normal appearance and thickness of the overlying epithelium (Fig. 3b). BRLH lesions tend to vary on HR-OCT, reflecting variable levels of cellular infiltration of the subepithelial tissue. HR-OCT may demonstrate homogenous lesions containing dot-like infiltrates as in CL or hyperreflective subepithelial tissue. The hyperreflective monomorphic infiltrates seen in BLRH correspond to a paucicellular infiltrate [77]. BRLH lesions with high levels of cellular infiltrates may also demonstrate hyporeflective subepithelial lesions with discrete borders. Although useful, HR-OCT alone cannot distinguish BRLH from CL.

### Diagnosis

The differential diagnosis for BRLH includes conjunctival papilloma, CA, chronic follicular conjunctivitis, foreign body granuloma, and CL [101]. BRLH requires a biopsy to confirm diagnosis and exclude malignancy. It exhibits a clonal proliferation and is characterized by well-defined lymphoid follicles with prominent germinal centers and a predominantly mature lymphocytic population [99]. Lymphoid follicles express reactivity to BCL-6 and CD10. The oncoprotein BCL-2 is not found in the follicular centers of BRLH, unlike in FL [102]. Flow cytometry may demonstrate polyclonal markers (CD3, CD5, CD20, CD23), although this is nonspecific and is also seen in CL [103, 104]. Immunohistochemistry reveals low levels of Ki-67, which is a marker of active cell proliferation [103].

### Management

In asymptomatic cases, patients may be managed with careful observation alone. In cases requiring treatment, most patients are treated with surgical excision and oral corticosteroids [95]. Surgery may be followed by adjuvant perilesional steroid injection, cryotherapy, or EBRT. To ensure that no malignant transformation has occurred, a repeat biopsy at 6–12 months is recommended, with annual follow-up and re-biopsy if changes are detected. Other medications, including doxycycline and cyclosporine, have been described in the literature as effective therapies, although evidence is limited to case reports [95, 98].

### Prognosis

BRLH is benign, but the most severe complication is progression to CL. This transformation is a rare occurrence, with few reports in the literature [74, 76]. In a series of 235 reported cases, only 2 demonstrated malignant transformation [95]. Recurrence of the lesion may occur in 20–30% of cases, but the overall prognosis for BRLH is favorable [101].

## Conjunctival Myxoma

### Disease Entity

Myxomas are benign connective tissue tumors derived from primitive mesenchyme [105, 106]. CMxs are thought to arise from Tenon’s capsule and occupy the substantia propria [105]. CMxs primarily occur independently as a localized disease process; however, they may arise in association with certain syndromic conditions, including Carney complex and Zollinger-Ellison syndrome [105–109].

### Epidemiology

CMxs are a rare entity, accounting for between 0.06 and 0.16% of all conjunctival lesions [106, 109, 110]. The mean age of presentation is 45.1–47.6 years with a 53.7–61.5% male predilection [106, 111]. No racial predisposition has been identified [106].

### Risk Factors

No specific risk factors have been identified for isolated CMxs. Personal or family history of Carney complex, an autosomal dominant syndrome, or its associated mutations in the *PRKAR1A* gene located on chromosome 17q24.2-3 (CNC1) and chromosome 2p16 (CNC2) may predispose patients to CMx [108]. Personal history of Zollinger-Ellison syndrome or personal or family history of inherited disorders associated with Zollinger-Ellison syndrome, including multiple endocrine neoplasia (MEN) type 1, neurofibromatosis type 1, and tuberous sclerosis, may also predispose patients to the development of CMxs [112].

### Clinical Presentation

**Fig. 4:**
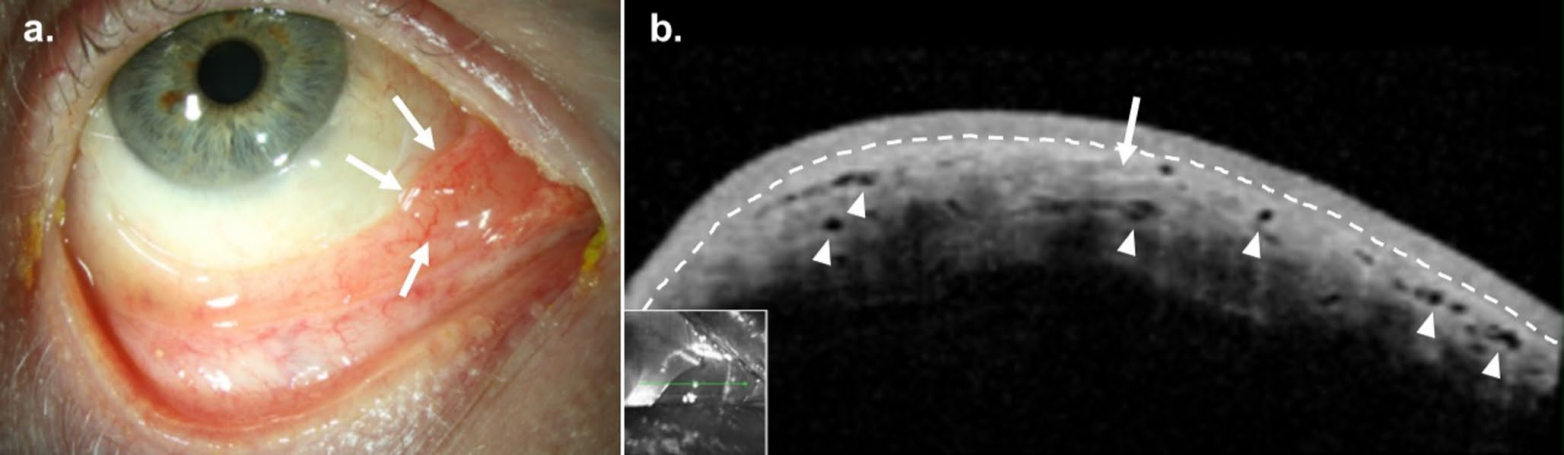
Conjunctival Myxoma. (**a**) Slit lamp examination of the right eye of a 67-year-old White male with a history of conjunctival myxoma status post surgical debulking revealed a residual salmon-colored conjunctival lesion along the nasal inferior fornix (arrows). (**b**) High resolution optical coherence tomography (HR-OCT) revealed a heterogeneous subepithelial lesion with medium to high reflectivity, hyperreflective linear features (arrow), and hypo-reflective intervening spaces (arrowheads) with a slightly hyperreflective, normal thickness overlying epithelium. The border between the epithelium and subepithelial space is labeled (dashed line). Findings were consistent with, and previous biopsy confirmed the diagnosis of conjunctival myxoma. Inset shows scan location

CMxs typically present as slow-growing, well-circumscribed, dome-shaped masses ranging in size between 1 and 30 mm in diameter and resembling conjunctival cysts (Fig. 4a) [106, 109, 111]. Lesions may appear white, yellow, pink, pale, or fleshy in color [106, 109, 111]. Clinically they may appear as conjunctival cysts, chemosis, or reminiscent of CL. CMxs may be semi-encapsulated or unencapsulated tumors [111]. Lesions are mobile and semitranslucent to fully translucent [106, 109, 111]. Lesions are generally rubbery or gelatinous in texture [106, 111]. 98.7% of cases present unilaterally [106]. CMxs are located on the temporal conjunctiva in 49% of cases, nasally in 27% of cases, and elsewhere on the conjunctiva in 24% of cases [111]. CMxs are generally asymptomatic but may cause redness and pain in rare cases [106]. Given their clinical presentation, CMxs may be mistaken for conjunctival cysts, chemosis, amelanotic nevi, amelanotic CM, or CL.

### HR-OCT

HR-OCT of CMx lesions demonstrate a heterogeneous subepithelial mass with mixed hypo- and hyperreflectivity lined by a highly hyperreflective band (Fig. 4b) [105, 113]. The subepithelial mass may contain round or linear hyporeflective cavities that correspond to blood vessels [113]. The overlying epithelium appears normal to slightly increased thickness with normal reflectivity [105, 113]. Large lesions may demonstrate posterior shadowing [105].

### Diagnosis

Histopathologic evaluation is required for definitive diagnosis of CMxs [106, 114]. Histopathologic evaluation of a CMx demonstrates distinct mucinous, fibrillary, and stromal cellular components [105]. The mucoid material constitutes the majority of the tumor volume and is composed of hyaluronic acid and chondroitin sulfate with scarce interspersed spindle- and stellate-shaped cells, vascular structures, and inflammatory cells (i.e. lymphocytes, macrophages, and mast cells) [105, 109, 111]. The fibrillary component is composed of a meshwork of reticulin fibers or, less frequently, ropey collagen [105, 106, 111]. The mucinous component is slightly basophilic and stains positively with Alcian blue with dissolution of color when treated with hyaluronidase [105, 106, 114]. The stromal cellular component stains positively with mucicarmine and colloidal iron [111]. Periodic acid-Schiff, oil red O, and Bodian stains are negative [111]. Immunohistochemistry demonstrates positivity for vimentin, RB1, nuclear anti-Ki67, CD34, and CD68 and negativity for SMA, SOX10, S100, desmin, and GLUT1 [106, 109, 114]. HR-OCT may be helpful in distinguishing CMxs from similar-appearing conjunctival lesions [105]. On ultrasound biomicroscopy, CMxs demonstrate dome-shaped subepithelial masses with homogenously low internal reflectivity and hypoechogenic foci scattered throughout representing vascularization [115]. CMxs have not been reported to invade the sclera or orbit [115].

### Management

The primary treatment for CMxs is complete surgical excision [106, 109, 111, 114]. In addition, cryotherapy may be applied at the time of surgery [106]. Given the adverse outcomes of the syndromic conditions associated with CMxs, patients should be referred for systemic evaluation [111]. Specifically, echocardiography should be conducted in patients with suspected Carney complex, as embolic events related to cardiac myxomas are the primary cause of morbidity and mortality in this condition [108].

### Prognosis

The systemic prognosis of CMx is generally very good as there have been no reported cases of malignant transformation and the rate of recurrence is 2.6% [106, 109, 111]. There are no reported cases of death due to CMx.

## Conjunctival Neuroma

### Disease Entity

CNs are extremely rare, benign nerve sheath tumors originating from the Schwann cells, connective tissue, and axons of nerves coursing through the substantia propria. CNs are another example of subepithelial lesions associated with a systemic syndrome. CNs almost exclusively arise in the context of MEN type 2b (MEN2b) as a result of a gain-of-function mutation in the RET tyrosine kinase receptor that is highly expressed in neural crest and neuroendocrine tissues [116]. The RET proto-oncogene is located on chromosome 10q11.2 [116]. The two most common mutations, a M918T mutation in exon 16 and an A883F mutation in exon 15, account for over 95% of cases of MEN2b [117]. Rarely, CNs may occur as the result of a gain-of-function frameshift mutation in exon 20 of the gene encoding the SOS1 guanine nucleotide-exchange factor in “pure mucosal neuroma syndrome” (MNS), though this is a diagnosis of exclusion [118].

### Epidemiology

CNs are exceedingly rare subepithelial lesions, as neural tumors of all variety (including neuromas, neurofibromas, and schwannomas) account for 0.06% of all amelanotic conjunctival tumors [110]. Further, CNs occur almost exclusively as a result of MENb which is a rare syndrome in its own right with an estimated prevalence of between 1 in 600,000 to as low as 1 in 39 million [116, 117]. CNs have been reported to occur in between 79 and 87% of patients with MEN2b [119, 120]. The prevalence of MNS has not yet been established, as this is a recently described entity.

### Risk Factors

The primary risk factor for the development of a CN is a personal or family history of MEN2b or MNS. Approximately half of the cases of MEN2b are the result of autosomal dominant inheritance and the remaining half are due to de novo germline mutations [121, 122]. The mode of inheritance of MNS is believed to be autosomal dominant; however, given the recent characterization of the syndrome, further investigation is required to draw a definitive conclusion [118].

### Clinical Presentation

**Fig. 5:**
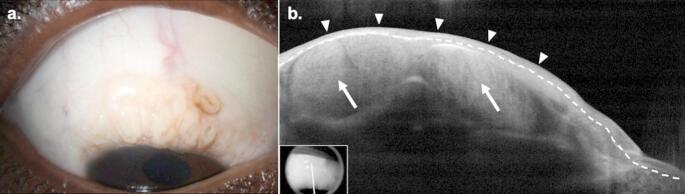
Conjunctival Neuroma. (**a**) Slit lamp examination of the left eye of an 18-year-old Black female with multiple endocrine neoplasia type 2b revealed a superior elevated globular conjunctival lesion with a serpentine appearance. (**b**) High resolution optical coherence tomography (HR-OCT) revealed a subepithelial lesion of mixed reflectivity with lobular components (arrows) and a hyperreflective overlying epithelium with normal thickness (arrowheads). The border between the epithelium and subepithelial space is labeled (dashed line). Findings were consistent with, and biopsy confirmed diagnosis of mucosal neuroma. Inset shows scan location

CNs typically present as an elevated, gelatinous subconjunctival nodule or wormlike mass with associated prominent nerves (Fig. 5a). The lesion may demonstrate surrounding neovascularization. Associated symptoms may include keratoconjunctivitis sicca, superficial punctate keratitis, and decreased corneal sensation due to decreased tear production, autonomic dysregulation, and neurotrophic keratopathy [123]. Rarely, CNs may result in secondary glaucoma [124, 125].

In the context of MEN2b, patients also display prominent corneal nerves in 100% of cases, eyelid neuromas or thickening in 80–88% of cases, prominent perilimbal blood vessels in 40% of cases, multicentric medullary thyroid carcinoma in almost 100% of cases (over 80% within the first year of life), bilateral, benign pheochromocytoma in 30–50% of cases, other mucosal neuromas (e.g. oral or gastrointestinal), marfanoid habitus, musculoskeletal abnormalities (e.g. slipped femoral epiphysis, joint laxity, kyphoscoliosis, or lordosis), and abnormal facies characterized by a long, narrow face with a wide-eyed expression, broad-based nose, and thickened, nodular lips [116, 117, 119–121].

In the context of MNS, patients may also present with prominent corneal nerves, other mucosal neuromas, marfanoid body habitus, and gingival hypertrophy, though the complete phenotypic spectrum has not yet been elucidated [118]. Importantly, MNS is distinct from MEN2b in that MNS patients do not develop the endocrinopathies typically associated with MEN2b [118].

### HR-OCT

HR-OCT of CN lesions demonstrate subepithelial masses of mixed reflectivity with lobular and whirling cellular features (Fig. 5b). The overlying epithelium is normal in thickness but may show hyperreflectivity [125, 126].

### Diagnosis

Patients with suspected CN should undergo thorough ophthalmic examination, including measurement of intraocular pressures, slit lamp examination, and, if the lesion is located near the corneoscleral limbus, gonioscopy. Histopathologic evaluation following incisional or excisional biopsy is required for definitive diagnosis of CNs. Histopathologic evaluation of a CN demonstrates a well-circumscribed, unencapsulated mass composed of enlarged neural bundles containing spindle-shaped cells with tapering nuclei within the substantia propria [127, 128]. Neural bundles are comprised of Schwann cells and poorly myelinated axons [126, 129]. CNs demonstrate roughly equal proportions of neurofilament-positive axons and S100-positive Schwann cells with positive epithelial membrane antigen stain at the tumor periphery on immunohistochemistry [125, 127, 128]. HR-OCT can help differentiate CNs from other subepithelial lesions and, in some cases, obviate the need for biopsy [125, 126]. In vivo confocal microscopy demonstrates thickened, hyperreflective, disorganized bundles of subepithelial conjunctival nerves with prominent loops, dilations, and bifurcations; however, it is generally not employed in clinical practice [123].

### Management

CNs are benign and generally do not require treatment, though they may be surgically excised if they are large, cause discomfort, or are associated with secondary glaucoma [125, 130]. Plexiform lesions may require multiple debulking procedures, thereby increasing the risk of scarring and symblepharon [130]. Identification of a CN, especially in the presence of prominent corneal or conjunctival nerves, should raise suspicion for MEN2b. These patients should be referred for further genetic, endocrine, and oncologic workup. If a genetic mutation is identified, family members should undergo screening to ensure timely prophylactic medical and surgical intervention [117].

### Prognosis

The systemic prognosis of solitary CN is very good as malignant transformation is extremely rare [130]. Though the ophthalmic manifestations of MEN2b generally do not affect vision, recognition can allow for early diagnosis and treatment of MEN2b-associated neoplasms, the major cause of morbidity and mortality in these patients [116, 122]. The long-term outcomes of MNS have not been established [127, 128].

## Conjunctival Amyloidosis

### Disease Entity

Amyloidosis refers to a heterogeneous group of disorders characterized by the synthesis and deposition of insoluble fibrillary aggregates of misfolded proteins leading to progressive organ damage [131, 132]. At least 42 unique types of amyloid fibril protein have been identified that variably affect different end organs [131, 132]. Amyloid deposition may occur extensively, resulting in systemic disease, or focally, resulting in localized disease [132]. CA is a primarily localized form of amyloidosis that is most commonly associated with AL lambda and AL kappa amyloid fibril protein [133–135].

### Epidemiology

CA is a rare entity, accounting for between 0.20 and 0.37% of all conjunctival lesions [110, 136]. CA is predominantly a localized process (88% of cases), though it may occur in response to a preceding local conjunctival disorder (termed “reactive” or “secondary” amyloidosis; 6% of cases) or as manifestation of primary systemic amyloidosis (6% of cases) [137]. The demographic characteristics of patients with localized, non-systemic CA appear to differ from those with primary systemic amyloidosis. The mean age of presentation is 48–55 years with a 60–63% female predilection in localized, non-systemic CA versus 65 years and 65% male predilection in primary systemic amyloidosis [137, 138].

### Risk Factors

There are two known risk factors for primary AL amyloidosis, including pre-existing monoclonal gammopathy and specific identified single nucleotide polymorphisms (e.g. rs9344 in the *CCND1* splice site and rs79419269 near the *SMARCD3* gene) [139, 140]. Secondary CA may occur in response to deposition of serum protein A, an acute phase reactant [137]. As such, local inflammation (e.g. trachoma, strabismus surgery, or allergic conjunctivitis) is a risk factor for the development of secondary CA [137, 141, 142]. Additionally, there are reports of secondary CA in association with systemic inflammatory conditions, including lymphoma, rheumatoid arthritis, Churg-Strauss syndrome, syphilis, hyperglobulinemia, hypothyroidism, and Addison disease [137].

### Clinical Presentation

**Fig. 6:**
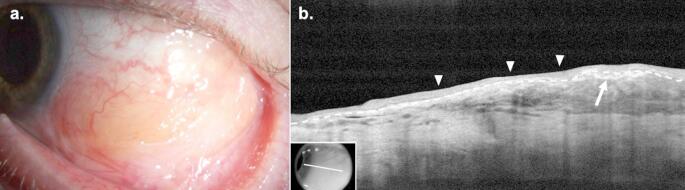
Conjunctival amyloidosis. (**a**) Slit lamp examination of the left eye of a 65-year-old White male revealed a pink and yellow-orange, fleshy conjunctival lesion extending inferotemporally into the fornix and left lower eyelid. The patient was initially thought to have conjunctival lymphoma based on clinical appearance. (**b**) High resolution optical coherence tomography (HR-OCT) revealed a subepithelial, heterogeneous conjunctival infiltrate with irregular borders, hyperreflective linear opacities (arrow), and a hyperreflective overlying epithelium with normal thickness (arrowheads). The border between the epithelium and subepithelial space is labeled (dashed line). Findings were consistent with, and biopsy confirmed the diagnosis of amyloidosis. Inset shows scan location

CA typically present as confluent fusiform lesions or polypoidal papules that are yellow- to salmon-colored (Fig. 6a) [137, 138]. Such lesions are present in 63–84% of cases [137, 138]. Given their clinical presentation, CA may be erroneously mistaken for CL. Lesions are localized masses or diffuse across the conjunctiva in 36% and 64% of cases, respectively [137]. The bulbar conjunctiva is involved in 31–34% of cases, the palpebral conjunctiva is involved in 25–52% of cases, the fornix is involved in 3% of cases, and lesions involve multiple locations in 8–14% of cases [137, 138]. CA is unilateral in 60–62% of cases and bilateral in 38–40% of cases [137, 143]. Other associated manifestations include subconjunctival hemorrhage in 23–33% of cases, blepharoptosis in 25–30%, thickened palpebral conjunctiva in 14%, pain in 6%, epiphora in 6%, pseudoptosis in 5%, redness in 3%, decreased visual acuity in 2%, and ectropion in 2% [137, 138]. In patients with subconjunctival hemorrhages, between 40 and 67% are recurrent [137, 138]. Propensity for subconjunctival hemorrhage can be attributed to blood vessel fragility secondary to amyloid deposition [133, 137, 144].

### HR-OCT

HR-OCT of CA lesions demonstrate a heterogenous, hyporeflective subepithelial mass with irregular borders containing linear hyperreflective opacities corresponding to amyloid crystal deposition (Fig. 6b) [77]. The overlying epithelium appears normal in thickness and may have normal or hyperreflectivity [30].

### Diagnosis

Patients with suspected CA should undergo thorough ophthalmic examination; however, definitive diagnosis can only be made with histopathologic evaluation following incisional or excisional biopsy. Histopathologic evaluation of CA demonstrates several pathognomonic features. On H&E staining, amyloid appears homogeneously eosinophilic with a granular and filamentous texture [131, 137]. On crystal violet, amyloid stains metachromatically [131]. Amyloid binds with high affinity to Congo red dye and demonstrates apple-green, yellow, or orange birefringence and dichroism when viewed under polarized light microscopy [131, 137]. Immunohistochemistry corresponds to the amyloid precursor protein. Patients with CA may demonstrate monoclonal lambda and kappa light chains on immunohistochemical staining [133, 137, 141, 143–145]. In cases of secondary CA, immunostaining may demonstrate the presence of serum protein A [145]. Though generally not performed in clinical practice, transmission electron microscopy demonstrates rigid, non-branching fibrils 7.5–10 nm in diameter [131]. Similarly, amyloid produces a pattern of bundles of β-sheet fibrillary proteins on X-ray diffraction [131]. A recent investigation by Pilotte et al. has established the feasibility of using an amyloid-binding fluorescent tracer molecule, AMDX-9101, to identify conjunctival amyloidogenic transthyretin (TTR) deposition in TTR amyloidosis, though studies are needed to characterize the safety and efficacy of this novel diagnostic modality in vivo [146]. Identification of the amyloid protein subtype may help to guide systemic workup. As above, HR-OCT is an easy-to-use and practical clinical tool that may help to guide diagnosis.

### Management

Management of CA varies depending on the extent of the lesion and the presence of systemic involvement. CA may be managed conservatively with observation, ocular lubrication, and bandage contact lenses if the patient remains comfortable and there are no signs of disease progression [133, 137]. In symptomatic patients, the primary treatment for CA is surgical excision or debulking [133, 137, 143]. Surgical intervention may be complicated by tissue friability and hemorrhagic tendency [138, 143]. Cryotherapy may serve as an alternative or adjunct therapy for CA [133, 147]. Uncommonly, attempts have been made to treat CA with CO_2_ laser vaporization and EBRT; however, these are not standard of practice and further investigation is necessary to determine safety and efficacy [143, 148, 149]. Patients with CA should be referred to an internist for systemic workup to rule out systemic disease, including rheumatologic disease and plasma cell dyscrasias [150].

### Prognosis

The rate of recurrence following surgical excision of localized, non-systemic CA is reported to be between 15 and 23% [137, 143, 144]. There are no reported cases of death due to localized, non-systemic CA. The morbidity and mortality of systemic amyloidosis varies with disease stage and the extent of organ involvement [151].

## Conclusions

Subepithelial lesions of the ocular surface pose a significant challenge diagnostically, as many of these pathologies are uncommon and some can mimic other clinical entities. Many of these subepithelial lesions may lead to or occur as a result of systemic disease. The development of novel diagnostic tools, such as HR-OCT, has revolutionized the evaluation of ocular surface lesions. HR-OCT is a valuable tool that can help to guide clinicians in their diagnosis and management of subepithelial conjunctival tumors, though it is important to note that HR-OCT cannot entirely replace histopathologic analysis. Additionally, though the standard treatment for many lesions remains surgical excision, recent advancements have introduced alternative treatment options, such as CPI in CM, for patients who are poor surgical candidates. Future investigations, diagnostic modalities, and interventions will likely contribute to less invasive, targeted diagnostics and therapies in the management of subepithelial conjunctival lesions.

## Human and Animal Rights

All reported studies/experiments with human or animal subjects performed by the authors have been previously published and complied with all applicable ethical standards (including the Helsinki declaration and its amendments, institutional/national research committee standards, and international/national/institutional guidelines).

## Key References

- Zhu T, Zong C, Li Y, Jia S, Shi H, Tian H, et al. High-risk histopathologic features in local advanced conjunctival melanoma. Acta Ophthalmol. 2024;102(5):e851-e61. 10.1111/aos.16662
-
  - This comprehensive retrospective study identifies the histopathologic features associated with poor outcomes in conjunctival melanoma.
- Zeiger JS, Lally SE, Dalvin LA, Shields CL. Advances in conjunctival melanoma: clinical features, diagnostic modalities, staging, genetic markers, and management. Can J Ophthalmol. 2024;59(4):209 − 17. 10.1016/j.jcjo.2023.02.003
-
  - This manuscript is noteworthy because it provides an update on the clinical features, diagnostic modalities, AJCC staging, genetic markers, and management of conjunctival melanoma.
- Sa HS, Daniel C, Esmaeli B. Update on Immune Checkpoint Inhibitors for Conjunctival Melanoma. J Ophthalmic Vis Res. 2022;17(3):405 − 12. 10.18502/jovr.v17i3.11579
-
  - This important review article provides a comprehensive summary of the current literature regarding the use of immune checkpoint inhibitors for the treatment of conjunctival melanoma.
- Pinnix CC, Dabaja BS, Gunther JR, Fang PQ, Wu SY, Nastoupil LJ, et al. Response-Adapted Ultralow-Dose Radiation Therapy for Orbital Indolent B-Cell Lymphoma: A Phase 2 Nonrandomized Controlled Trial. JAMA Oncol. 2024;10(9):1195 − 203. 10.1001/jamaoncol.2024.2112
-
  - This prospective phase 2 clinical trial is important as it demonstrated an excellent response to treatment of conjunctival lymphoma using ultralow-dose radiation therapy (4 Gy in 2 fractions).

## Data Availability

No datasets were generated or analysed during the current study.
